# Impact of a multifaceted intervention on the incidence of pressure ulcers in a medical-surgical icu. a before-after study

**DOI:** 10.1186/2197-425X-3-S1-A926

**Published:** 2015-10-01

**Authors:** CI Loudet, MC Marchena, R Maradeo, S Fernández, V Romero, G Valenzuela, M Ramírez, S Rojas, LI Tumino, AL González, R Reina, E Estenssoro

**Affiliations:** Hospital General San Martín de La Plata, La Plata, Argentina

## Introduction

Quality management to improve healthcare processes is a priority in the ICU. In a previous study, we detected a high incidence of Pressure Ulcers (PUs) including a notable amount of advanced-grade PUs.

## Objectives

To determine the effectiveness of a multidisciplinary improvement program to reduce and manage PUs in critical care patients.

## Methods

Quasi-experimental before-after study in a 14-bed medical-surgical ICU in Argentina. Consecutive patients on mechanical ventilation (MV) >3 days, over a pre- and a post-intervention (Pre-I and Post-I) period were included. In each period, we recorded: epidemiological variables, reason for admission, severity-of-illness score on admission (APACHE II and SOFA_24_), duration of MV and ICU stay, nurse-to-patient ratio and ICU mortality.

### Interventions

A "Process Improvement" team was formed, comprised of nurses, dermatologists and intensivists. The team designed a multifaceted intervention consisting of educational sessions, prevention and monitoring PU checklist, a telemedicine tool (*WhatsApp*^®^ group) for monitoring and decision-making, and a “family prevention bundle” (participation in monitoring and hydration-massages).

### Outcome measures

Performance measurement indicators (Table 1) of prevention and management of PUs were selected and calculated in the pre and post-intervention periods.

Data were analyzed according to their nature. A logistic regression analysis was used to identify independent factors related to the development of advanced-grade PUs. A p value < 0.05 was considered significant.

## Results

In the Pre-I and Post-I periods, 55 and 69 patients were admitted to the ICU; age 47 ± 18, 39 ± 17 (p: 0.01); APACHE II 18 ± 7, 18 ± 6 (p: 0.77); SOFA_24_ 7 [4-9], 8 [6-10] (p: 0.06), MV-days 17 [9-46], 14 [8-34] (p: 0.49); ICU days 19 [8-47], 19.5 [11-36] (p: 0.42); nurse-to-patient ratio 1:2.5, 1:2.4 (p: 0.86), respectively. ICU mortality was 23(42%) and 24 (35%) (p: 0.42).

Pre-I and Post-I indicator performance are shown in Table 1. In the logistic regression model, duration of MV and SOFA_24_ were positively associated with the outcome of advanced-grade PUs (OR 1.04; CI95% 1.01-1.07 and OR 1.43; CI95% 1.14-1.79 respectively), while the multifaceted educational intervention acted as a protective factor (OR 0.04; CI95% 0.09-0.19). Adjustment and discrimination of the model were appropriate (Hosmer-Lemeshow test: 3.71 (p = 0.86); and AUCROC 0.88; CI95% 0.81-0.96).

**Table 1:** Pressure ulcer indicator performance

	Pre-Intervention period (N 55)	Post-Intervention period (N 69)	p value
Braden Scale*	3 [3-4]	3 [3-4]	0.87
**Use of pressure prevention mattress**	26 (48)	59 (85)	< 0.01
**Family prevention bundle**	5 (9)	39 (57)	< 0.01
**Pressure ulcer incidence**	41 (75)	37 (54)	< 0.05
**Advanced-grade PU**	27 (49)	7 (10)	< 0.01
**Days to develop PU**	3 [3-5]	9 [6-20]	< 0.01
**PU at discharge**	38 (69)	18 (26)	< 0.01
*PU Risk 1: mild 2: moderate 3: high 4: very high			
**Data presented as N (%) and median [p25-75]**			

## Conclusions

It was possible to implement a multifaceted educational intervention with a group composed of intensivists and nurses, along with dermatologists and the voluntary participation of patient family members. This intervention was independently associated with a reduction in advanced-grade pressure ulcers.

